# CRUISE, a Tool for the Detection of Iterons in Circular Rep-Encoding Single-Stranded DNA Viruses

**DOI:** 10.1128/mra.01123-22

**Published:** 2022-12-01

**Authors:** Adam Jones, George W. Kasun, Joel Stover, Kenneth M. Stedman, Ignacio de la Higuera

**Affiliations:** a Department of Biology, Center for Life in Extreme Environments, Portland State University, Portland, Oregon, USA; b Westview High School, Portland, Oregon, USA; c AbSci, Vancouver, Washington, USA; DOE Joint Genome Institute

## Abstract

Iterons are short, repeated DNA sequences that are important for the replication of circular single-stranded DNA viruses. No tools that can reliably predict iterons are currently available. The CRUcivirus Iteron SEarch (CRUISE) tool is a computational tool that identifies iteron candidates near stem-loop structures in viral genomes.

## ANNOUNCEMENT

**FIG 1:**
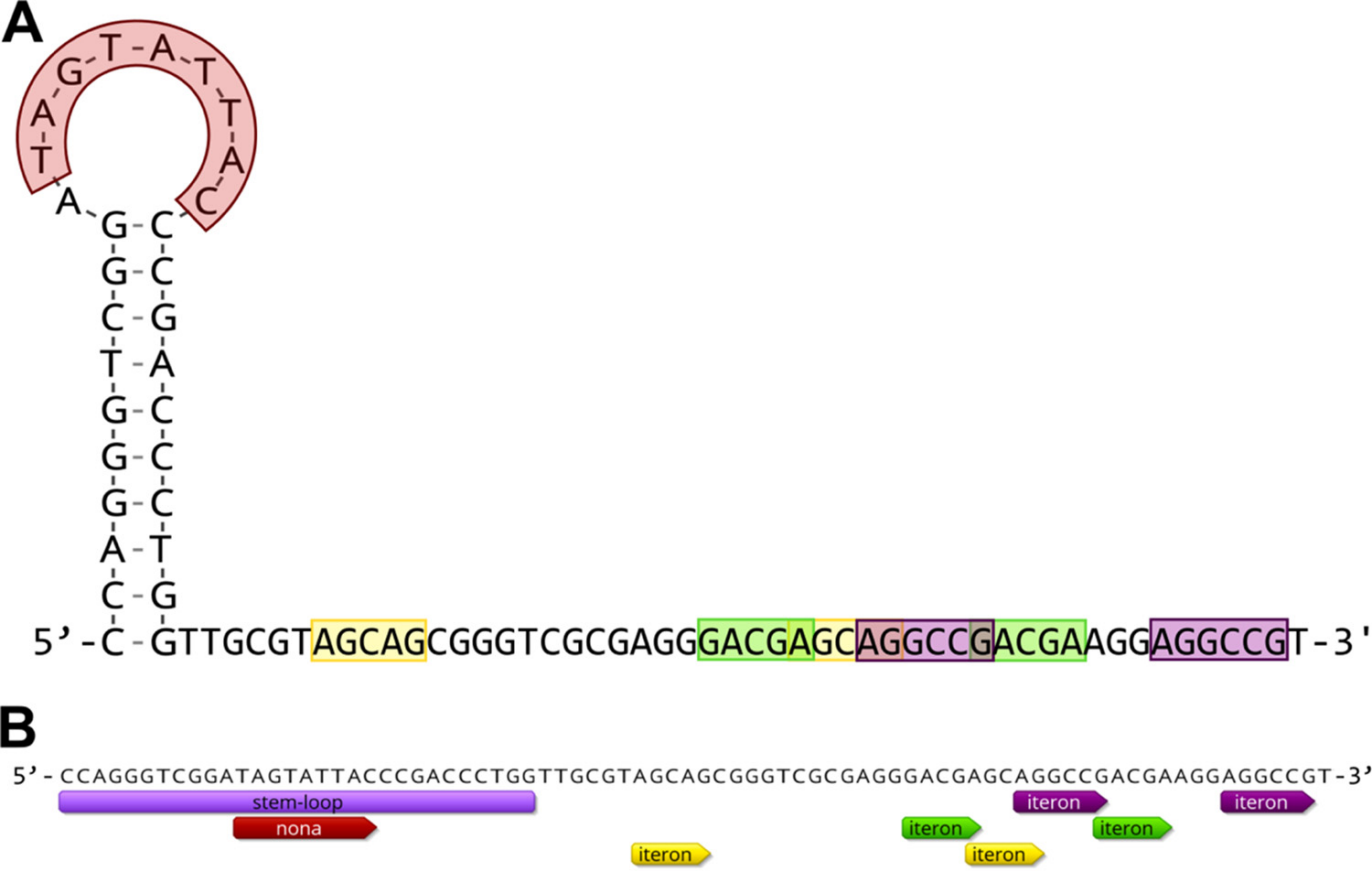
(A) Diagram of the predicted origin of replication of CruV-249, including the stem-loop structure (with the conserved nonanucleotide motif shaded red) predicted by StemLoop-Finder (11), and 3 different iteron sets predicted by CRUISE. (B) Annotations generated by StemLoop-Finder (labeled stem-loop and nona) and by CRUISE (different iteron sets) for CruV-249, after the GFF3 output file was imported into Geneious Prime. Different iteron sets within the same genome were automatically labeled with different colors by the software.

Circular Rep-encoding single-stranded DNA (CRESS-DNA) viruses replicate via rolling circle replication (RCR) (1, 2). This process begins with the binding of the viral replication-associated protein (Rep) to the origin of replication in the viral double-stranded DNA (dsDNA) replicative intermediate, which is facilitated by iterons (3–5). Iterons are short sequence repeats near stem-loop structures at the genomes’ origin of replication (6) (Fig. 1A). Iterons are also hypothesized to constrain recombination events in CRESS-DNA viruses (7), which makes them particularly relevant to the analysis of chimeric genomes such as those of cruciviruses (8–10). Iteron identification is essential for the analysis of CRESS-DNA virus genomes and critical for the design of experiments aimed at understanding RCR processes. However, iterons have traditionally been found manually, because there are no publicly available tools to automate the process. The CRUcivirus Iteron SEarch (CRUISE) tool performs this task by efficiently and accurately identifying and ranking iteron candidates in CRESS-DNA virus genomes.

CRUISE is written in Python 3. It uses general feature format (GFF3) sequence files for both input and output, adding iteron annotations to already annotated genomes (Fig. 1B). CRUISE performs a text search to find exact direct repeats in the region of the genome near an annotated origin of replication (stem-loop and nonanucleotide motif, as predicted with StemLoop-Finder [11]). After a preliminary filter is used to remove invalid results, an additional search looks for inexact matches to current candidate iteron sets. CRUISE then ranks the sets of iteron candidates based on metrics such as repeat length, distances from the origin of replication and between repeats, base content, and similarity to known iterons. Ambiguous bases are not accounted for, because the additional variation would produce an overwhelming number of results. CRUISE can be launched from a command-line interface, and the parameters that influence the scoring algorithm can be customized to select for preferred iteron features. CRUISE can also use a database of previously discovered iteron sequences to find matches in the input set. The tool was designed for crucivirus genome analysis but is easily adapted to any CRESS-DNA genome.

CRUISE was applied to a database of 278 recently discovered crucivirus genomes with annotated origins of replication (9), and 584 iteron candidates were found in 262 genomes. When used on the same set of genomes with an iteron sequence database, CRUISE marked an additional 223 iteron candidates. Additionally, CRUISE was applied to a set of 35 circular DNA virus genomes that had previously been manually searched for iterons (12). All manually identified iteron candidates within the set parameters were automatically identified by CRUISE. Furthermore, CRUISE identified at least 1 novel iteron set in 23 of those genomes, many of which appear to be better candidates than the previously identified iteron sets.

To our knowledge, CRUISE is the only currently available iteron prediction tool. CRUISE provides a novel way to automate an important part of single-stranded DNA (ssDNA) genome annotation. This tool relies on stem-loop and nonanucleotide annotations, which can be provided by the sister program StemLoop-Finder (11). In combination, the two tools should provide researchers with a rapid and efficient way to predict CRESS-DNA virus origins of replication in genomic datasets.

### Data availability.

The software source code is available at a public GitHub repository (https://github.com/adamjnes/CRUISE). It will remain freely available for the next 10 years alongside instructions for use and any applicable updates.
